# Expensive seems better: The price of a non-effective drug modulates its perceived efficacy

**DOI:** 10.1186/s41235-023-00463-4

**Published:** 2023-01-26

**Authors:** Marcos Díaz-Lago, Fernando Blanco, Helena Matute

**Affiliations:** 1 Departamento de Psicología, Universidad de Deusto, Apartado 1, 48080 Bilbao, Spain; 2 Universidad de Granada, Granada, Spain

**Keywords:** Medical decision-making, Illusion of causality, Price, Drug efficacy, Alternative medicines

## Abstract

Previous studies have shown that the price of a given product impacts the perceived quality of such product. This finding was also observed in medical contexts, showing that expensive drugs increase the placebo effect compared to inexpensive ones. However, addressing a drug's efficacy requires making causal inferences between the drug and the healing. These inferences rely on the contingency between these two events, a factor that is difficult to control in the placebo research. The present study aimed to test whether the price of a given drug modulates its perceived efficacy using a proper (though fictitious) non-effective drug, so that not only the objective contingency, but also the probability of the cause and the probability of the effect could be adequately controlled for. We expected higher efficacy judgements for the expensive non-effective drug than for the inexpensive one. To test this hypothesis, 60 volunteers participated in a contingency learning task that was programmed so that 72% of the patients healed regardless of whether they took the drug. Approximately one-half of the participants were told that the drug was expensive, whereas the other half were told that it was inexpensive. As expected, the efficacy judgements of participants who saw the expensive drug were significantly higher than those who saw the inexpensive one. Overall, our results showed that the price of a non-effective drug modulates its perceived efficacy, an effect that seems to be mediated by the estimated number of doses administered. This result parallels findings in the placebo literature but using a laboratory methodology that allows stronger control of the variables, suggesting that the illusory overestimation produced by the more expensive treatments might be on the basis of the greater efficacy of the more expensive placebos.

## Introduction

The COVID-19 health crisis highlighted the importance of the consequences that our subjective judgements about the efficacy of a medicine can have (e.g. COVID-19 vaccines, Henry et al., 2021). Decisions in the medical context (like physicians selecting the most effective drug, patients choosing between alternative and scientific treatments, or parents deciding whether to vaccinate their children) could have a significant impact not only on the lives of those who make the decision, but also on the lives of others (Henry et al., 2021; Matute et al., 2011). One would expect that in such situations, all the information available should be carefully considered to make the best decision. However, evidence from psychological research shows that people are prone to use different heuristics in their decision-making process (Blumenthal-Barby & Krieger, 2015), including, as in other areas, the price–quality beliefs. Heuristic rules like this one that can drive decisions away from the rational norm, are present not only in laypeople, but also in experts, as research has shown (Neal et al., 2022). More specifically, research conducted with medical staff provides evidence of decision-making biases, which could be even larger in more experienced professionals (Mamede et al., 2010).

Psychologists and behavioural economists have demonstrated the influence of an item's price on its perceived quality (Monroe, 2003). From durable (e.g. laptops, cell phones) to non-durable goods (e.g. detergents, granola bars), price affects the consumers’ decision-making not only because it works as a quality cue (Olson, 1977) but also as a heuristic (Judd, 2000). Specifically, according to this price–quality heuristic, the perceived quality of a product would increase with price, especially if other information is lacking (Judd, 2000; Lichtenstein et al., 1993). Although the extent of this positive correlation between price and perceived quality varies, most recent systematic reviews and meta-analyses show that this effect is robust (Boyle & Lathrop, 2009; Völckner & Hofmann, 2007).

In addition, research focused on the effect of price on placebo shows larger effects in expensive treatments than in inexpensive ones, in domains from pain management (Waber et al., 2008), to motor functions in Parkinson's disease (Espay et al., 2015). Therefore, if we assume that the quality of a drug relies on its perceived effectiveness, these results would support the price–quality heuristic also in such a critical area as medical decision-making. However, addressing a drug's efficacy requires making causal inferences between the drug and the recovering, and these inferences rely on the contingency between these two events (Matute et al., 2015). Experts in the placebo and nocebo effects describe the “placebo response” as real health changes resulting from the intake of the inactive treatment, while the “placebo effect” refers to perceived changes that could be attributed to the patient’s expectations (Evers et al., 2018). In the experimental tradition that examines the effect of price on placebo effects, some researchers consider that the increase in the efficacy is not only perceived, because the placebo response ‘*alters the actual efficacy*’ of the product (Shiv et al., 2005, p. 391); while others assume that the actual efficacy does not vary (Evers et al., 2018). In either case, there are some critical variables related to the contingency between taking a drug and recovering that cannot be fully controlled in the placebo literature, as they are under the participant’s control. If people interpret that they healed, which is an ambiguous, subjective outcome that is difficult to control, this could affect the perceived probability of the outcome’s occurrence. This makes it hard to keep a null contingency in a placebo experiment in real life, thus making it impossible to conclude whether the price of the non-effective drug is inducing an illusory overestimation of causality or not.

To test the potential effect of the price on the perceived efficacy of a non-effective drug, we propose setting and controlling a laboratory scenario in which there is no contingency between the cause (i.e. taking the drug) and the effect (i.e. recovering/feeling better). Thus, we can control both the probability of the cause and the probability of the effect in this fictitious context, unlike experiments in more realistic settings. According to the most commonly used measure of contingency, the ΔP index (Allan, 1980), in a non-contingent condition, the probability of the effect (recovering/feeling better) should be the same when the cause (e.g. medicine) is present and when it is absent (i.e. *p*[*E*|*C*] = *p*[*E*|no*C*]). Thus, the actual difference between these two probabilities should equal zero. Therefore, testing the effect of the price in this controlled setting using a medical contingency learning task could be particularly interesting. Furthermore, according to this Δ*P* index, contingency is not only modulated by the probability of the effect, *p*(E), but also by the probability of the cause, *p*(C). Research shows that a high probability of the cause leads to overestimations of causality even in non-contingent situations (Matute et al., 2015). Therefore, using a passive, observational learning task in which the participants are asked to simply observe the medical records of fictitious patients should allow us not only to fully control all variables that could lead to changes in the contingency (i.e. *p*(C) and *p*(E)), but also to emulate observational situations in which people learn from the experience or testimonies of other people, something which is common, for instance, in the use of pseudomedicines (e.g. *‘Try this, it worked for me!’)*. Understanding how the cues to assess the efficacy of a non-effective drug work could provide valuable information to implement public health strategies and policies.

Considering the potential relevance of this line of research, we experimentally tested whether the price of a given drug modulates its perceived efficacy in a non-contingent scenario, while controlling not only the objective contingency, but also the probability of the cause and the probability of the effect by using a binary outcome (healed/not healed) which is not subject to subjective interpretation and can simply be present or absent. To our knowledge, these variables cannot be controlled, at least simultaneously, in the placebo literature. Therefore, this should allow us to isolate the overestimation of the causal relationship (i.e. the illusion of causality) from a placebo response and the subjective response of feeling better. Furthermore, our study aims to contribute evidence on the variables that activate the expectancies that mediate the placebo effects, particularly on the price as a decision-making heuristic in the medical domain. If we assume that the quality of a drug relies on its efficacy, according to the price–quality relationship (Judd, 2000) we should expect a more positive assessment of expensive products. Therefore, using a fictitious and non-effective drug in two different groups, we hypothesised that participants exposed to the expensive drug would provide higher efficacy judgements.

## Method

### Participants

Sixty anonymous Spanish Internet users agreed to take part in the study (four were younger than 18, and therefore were excluded from the sample). The final sample consisted of 30 women and 26 men (mean age 31.30 years, range 18–60, *SD* = 12.26). All gave their informed consent before starting the experiment.

### Materials

We used a computer task implemented in HTML and JavaScript. The programme included the informed consent and the contingency learning task, which was a modified version of the allergy task (Matute et al., 2011).

### Procedure and design

Participants ran the experiment online on their computers, accessing the study from a link on our research group's website, which redirected to the HTML document of the experiment. Before starting the task, we presented the informed consent (see the ‘Ethics approval and consent to participate’ subsection). If the volunteers agreed to participate, the programme randomly assigned them to one of the two experimental groups.

In the instructions, we asked participants to imagine being a medical doctor who had to judge the efficacy of an experimental drug (cause) on a fictitious disease (effect) by merely observing several medical records of a series of fictitious patients. These records were presented in a trial-by-trial format, that is, one patient per trial. A total of 50 trials were presented.

The only experimental manipulation was the price of the medicine that participants had to assess. Therefore, participants were asked to judge exactly the same non-effective drug (i.e. same name, same efficacy), but it could be either inexpensive (2€) or expensive (230€), depending on the experimental group. To put the price of the medicine into perspective, the budget of the medical staff was set to 6000€ per month for both groups. After reading the instructions, the computer programme presented one by one the medical records of the 50 fictitious patients. Each medical record contained information about the fictitious patient: the disease that he/she was suffering (Lindsay syndrome for all patients) and the patient identifier (upper panel), and whether he/she had taken the drug (i.e. Batatrim) or not (middle panel). Then, participants were asked a predictive question about whether they believed the patient would improve (*'yes'* or *'no'*). This question was included to keep participants engaged in the task. Finally, once they responded, information about the actual recovery or not was shown (lower panel).

**Fig. 1:**
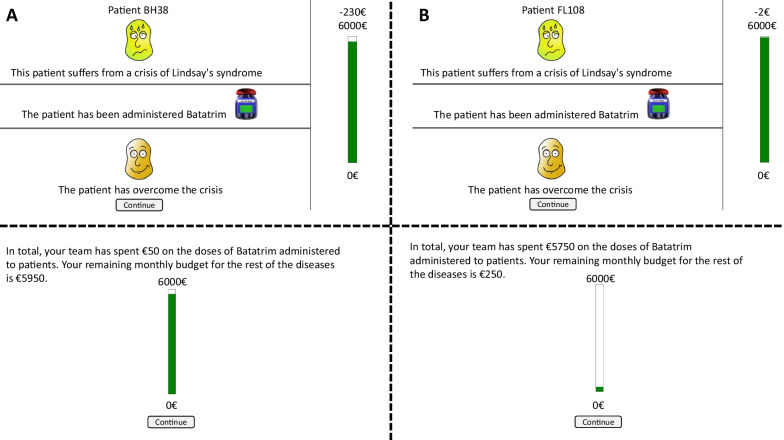
Examples of the individual trials in both the expensive (**A**) and inexpensive (**B**) groups (above), as well as the final budget (below)

Along with this information, located in the right-hand part of the screen, we also presented the updated remaining budget. Once participants saw the 50 medical records, we presented on a new screen the total amount of money spent on administering this drug and the remaining funds for other diseases. For the inexpensive group, the impact on the budget would be minor, while for the expensive one, almost all the money would be spent only on this medicine (see Fig. 1).

After collecting all this evidence, participants were asked to judge the relationship between taking the drug and recovering from the disease by answering the question *'To what extent do you think that Batatrim has helped to stop the crises of Lindsay Syndrome of the patients you have seen?'*. Participants could answer this question by clicking on a 0 to 100 scale (0 = *‘It has not helped at all’*; 50 = *‘It has been quite helpful’*; 100 = *‘It has helped perfectly’*). This judgement was the dependent variable in which we tested our hypothesis. Once participants judged the drug, we presented two additional questions to further explore (1) the influence of perceived efficacy and cost on the number of doses that they were willing to administer (*‘If it were up to you, given the efficacy and cost of the medicine, how many patients would you have given it to?’*), and (2) the estimation of doses given (*‘Out of the 50 patients, how many have been given the drug?’*).

Considering that the main goal of this study was to test the influence of the price on the perceived efficacy of a non-effective drug (therefore, controlling the placebo effect), the contingency between the drug (cause) and healing from the disease (effect) was set to zero for both experimental groups. Furthermore, we fully controlled the frequency of the cause and the frequency of the effect by setting the administration of the medicine to 50% of the fictitious patients, while the probability of recovery was identical regardless of whether the fictitious patients took the drug or not (72%). Thus, this was a truly non-contingent setting (Δ*P* = *p*(*E*|*C*) − *p*(*E*|no *C*) = 0.72–0.72 = 0) according to the Δ*P* index (Allan & Jenkins, 1980). We used a high percentage of patients recovering from the crises to favour the development of an illusion of causality and pseudoscientific health beliefs (Chow et al., 2019, 2021; Matute et al., 2015). The different trial types showing whether the medicine and the recovery were present or absent were randomly arranged (i.e. participants saw a random sequence of the trials, avoiding the problems of fixed sequences, such as recency effects).

## Results

**Fig. 2:**
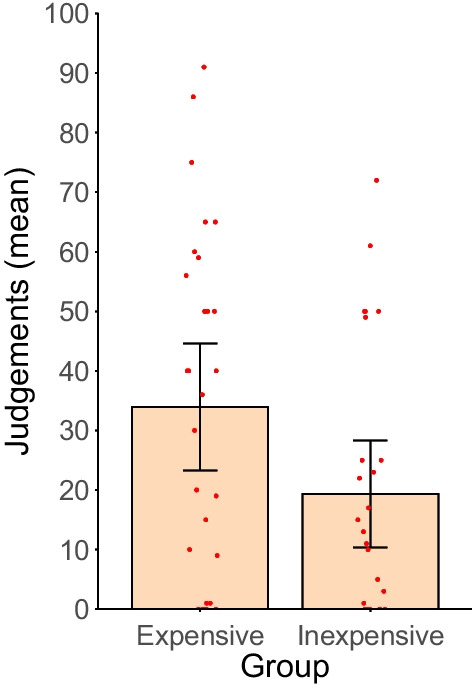
Judgements for the inexpensive and the expensive drug. *Note.* Bars represent the mean judgements for each group. Error bars depict the 95% confidence intervals for the means. Dots indicate the individual data of each participant

Figure 2 shows the mean judgements of the participants that saw an inexpensive drug compared with those that saw the expensive one. In line with previous findings in the contingency learning research (Chow et al., 2019; Matute et al., 2015), the judgements of both groups were not exactly accurate and departed from the actual contingency (zero). That is, both groups overestimated the efficacy of the drug to some extent. However, what is most interesting about the results depicted in Fig. 2 is that judgements differ as a function of the price of the medicine.

As expected, participants who observed patients taking an expensive drug seem to perceive it as more effective than those who observed the inexpensive drug. A t test for independent samples revealed a significant moderate effect (Cohen, 1988), *t*(54) = 2.117, *p* = 0.039, *d* = 0.567, 95% CI [0.014, 1.110].

In addition, we conducted further exploratory analyses to check the influence of the economic cost of the medicine on the participants' decision-making process. Specifically, we explore the role of two variables: (1) the number of doses that participants were willing to administer, and (2) the retrospective estimation of the number of doses administered through the experiment.

**Fig. 3:**
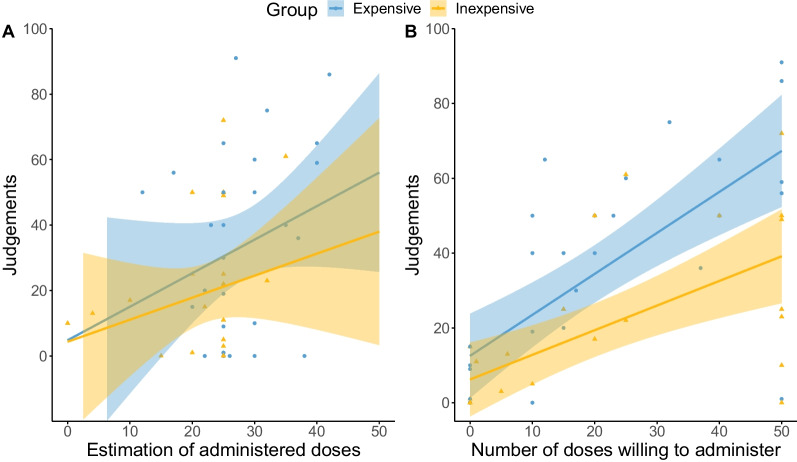
Scatterplots of the relationship between judgements and the estimation of **A** administered doses, and **B** the number of doses willing to administer. *Note.* Colours split experimental groups: yellow represents the inexpensive groups' responses, and blue represents the expensive group. Dots and triangles depict each participant's data, while the lines show the regression lines with 95% confidence intervals

Regarding the first variable, Student’s *t* test did not show a significant effect of the price of the drug in the number of doses participants would have been willing to administer if it had been up to them, *t*(54) = 0.066, *p* = 0.947. However, as shown in Fig. 3 (right), as the perceived efficacy of the drug increased, participants would have administered more doses if it had been up to them in both experimental groups. Pearson's coefficient revealed a large positive correlation, *r*(54) = 0.634, *p* < 0.001.

Figure 3 also depicts the relationship between the estimation of doses and the efficacy judgements (left). Pearson’s coefficient confirmed a significant medium positive correlation, *r*(54) = 0.339, *p* = 0.011. A Student’s t test on the estimation of doses administered revealed a modulation of price: participants in the expensive drug group reported to have seen significantly more patients taking the drug (*M* = 28.43, *SD* = 8.00) than those in the inexpensive drug group (*M* = 22.23, *SD* = 7.55), the effect of the price being large, *t*(54) = 2.969, *p* = 0.004, *d* = 0.796, 95% CI [0.220, 1.358].

**Fig. 4:**
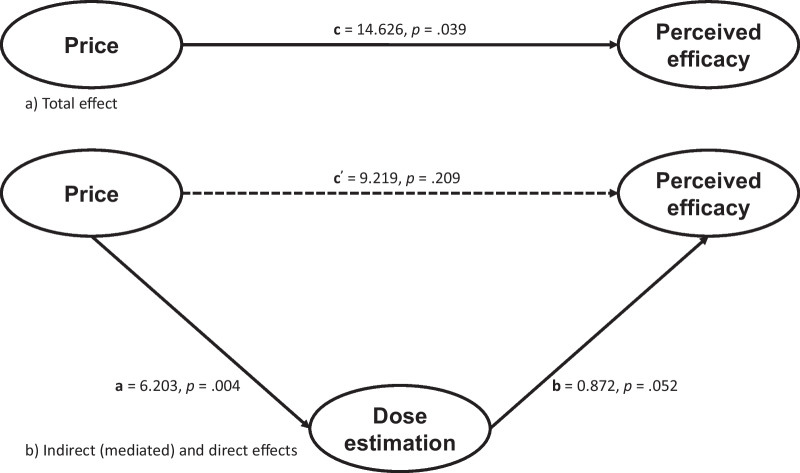
Structure and estimations of **a** the direct effect of the price on the perceived efficacy of a drug and **b** of the effect of the price on the perceived efficacy of a drug mediated by the estimation of administered doses

Considering this result, we explored the role of the estimation of the doses in the relationship of the effect of price on the perceived efficacy by conducting a mediation analysis using the *‘mediation’* package in R (Tingley et al., 2014). Figure 4 depicts the mediational structure and unstandardized path estimates.

As previously reported, the total effect of the price of the drug on the perceived efficacy (*c*) was significant, *t*(54) = 2.117, *p* = 0.039. However, when partitioned in two pathways to explore the role of the recall of the doses as a mediator variable, we can see a significant effect of the price of the drug on the estimation of the administered doses (*a*), *t*(54) = 2.969, *p* = 0.004, and a marginal significant effect of that estimation on the perceived efficacy of the drug (*b*), *t*(53) = 1.989, *p* = 0.052. Interestingly, when the mediator variable was included, the direct effect of the price on the perceived effectiveness of the drug (*c’*) was not significant, *t*(53) = 1.270, *p* = 0.209. To test if the mediation effects were statistically significant, we ran a bias-corrected bootstrap resampling method of 10,000 samples. The bootstrap revealed a significant total effect of the price on the effectiveness judgements, *c* = 14.626, *p* = 0.030, 95% CI [1.485,28.000], a significant indirect effect of the price on the effectiveness through the recall of the doses, *a* * *b* = 5.407, *p* = 0.018, 95% CI [0.585, 12.210] and, importantly, a non-significant direct effect of the price on the effectiveness when the recall of the doses was included as a mediator, *c′* = 9.219, *p* = 0.201, 95% CI [− 5.218, 23.380]. Overall, these results suggest that the estimation of the doses administered mediated the effect of the price on the perceived efficacy of the drug, although we should be cautious about drawing conclusions of these exploratory analyses and consider (1) that our study was not designed to test this mediational hypothesis, and (2) that the effect of the recall of the administered doses on the effectiveness judgements is marginally significant.

## Discussion

The present study aims to detect the effect of a drug's price on its perceived efficacy in a condition that warrants that the drug is completely ineffective. That is, the drug is non-contingent with the healing and both the probability of the cause and the probability of the effect are controlled for. Overall, the study's main results show some extent of overestimation in the perceived efficacy of the non-effective drug, regardless of its price. As previously mentioned, this is a common result in the contingency learning literature. Under certain conditions, when there is null contingency between the cause and the effect, but the probability of the recovery is high, people tend to systematically deviate their judgements from the actual contingency (zero), thus overestimating the relationship and giving rise to a bias called the illusion of causality. We programmed the task so that 72% of the fictitious patients recovered from the disease, irrespective of whether they had taken medicine. The high probability of the recovery (the effect), combined with a relatively low number of trials (i.e. patients), would have misled participants' when assessing the non-effective drug, hence replicating the illusion of causality effect (Chow et al., 2019; Matute et al., 2015; Shanks & Dickinson, 1987).

Interestingly, the statistical analysis confirmed that participants who saw the expensive drug judged it as significantly more effective, showing a stronger illusion, even though they saw exactly the same information as those in the inexpensive medicine group. This supports our hypothesis that the price of a non-effective, placebo-effect controlled (i.e. non-contingent) drug, modulates its perceived efficacy. More specifically, as expected by the price–quality relationship, the more expensive the drug, the higher the efficacy judgements. This result parallels findings observed in the placebo literature (Espay et al., 2015; Waber et al., 2008), but in a set-up in which the variables that affect the actual treatment-outcome contingency are fully controlled and the subjective interpretation of the occurrence of the outcome is not possible because it is clearly present or absent in each trial. In addition, the present results extend the effect of the price to observational situations, in which people do not learn about the efficacy of a drug through direct experience but through the observation of others, by fully controlling the frequency of the cause. Moreover, if we consider that the perceived quality of a drug lies in its perceived efficacy, our research provides further evidence on the price–quality relationship and supports Lichtenstein and colleagues’ claim that the higher the price of a product, the higher its perceived quality (Lichtenstein et al., 1993). This suggests that the price is used *‘as a surrogate for quality as a decision-making heuristic’* (Judd, 2000), also in medical contexts.

The exploratory analyses on the two additional questions about the number of doses that participants were willing to administer/thought were administered revealed interesting information. It is important to note that the probability of the cause is a critical variable that modulates the illusion of causality, even producing mediating effects in active tasks in which the administration of the drug is decided by the participants (Blanco et al., 2014). Specifically, the literature on causal learning shows that the higher the probability of the cause, the higher the overestimation of contingency (Matute et al., 2015). Therefore, those two final questions serve as indirect measures of (1) what the probability of the cause would have been if the task had allowed participants to decide the number of doses used, and (2) the participants' subjective perception of what the probability of the cause was.

First, we examine the willingness to administer more doses. In principle, being willing to administer more doses of a drug that is perceived as more effective is consistent with the idea that people will more often use products they consider of good quality. Therefore, this result informs us about how perceived efficacy would affect people’s behaviour in the future, in which they can enter into a vicious cycle of using more of a non-effective drug that they illusory believe it works and fuelling this belief by just using the drug more often. However, the number of doses that participants were willing to administer did not significantly differ between the expensive and inexpensive drug. This result challenges our price–quality heuristic hypothesis, since it would be expected that an expensive and, therefore, perceived as more effective drug (as the main result shows), would be used more often. The answer to this paradox seems to lie in the important role of another variable when assessing the drug’s efficacy: the retrospective estimation of the number of doses given.

The statistical difference between the expensive and inexpensive drug in terms of the recall of administered doses, and the overall correlation between that estimation and the efficacy judgements could help us better understand the effect of price on judgements. On the one hand, the more expensive the medicine is, the more often the participants believe that has been administered. On the other, the more often it appears to have been administered, the higher the illusory perception of efficacy. These two results suggest that the effect of the price in the perceived efficacy of a non-effective drug is not direct but mediated by the perceived probability of the cause. Although we should be cautious about drawing strong conclusions about a *post hoc* mediation analysis that has not yielded strong results, it seems to support the hypothesis that the price of the drug distorts the recall of the number of doses administered to the patients, which ultimately would be the factor that would modulate the perceived efficacy. More specifically, the more expensive the medicine is, the more often it seems to have been administered, leading to a higher illusory perception of efficacy. We can only hypothesise about why participants who saw an expensive medicine believed to have observed more patients that took the drug. One possibility is that the bar with the available budget was clearly reduced on each trial in the expensive drug, compared to the smaller reduction in the inexpensive group. This observation could lead participants to infer that, because the budget was substantially reduced, there were many drug doses administered (i.e. they believe that there were many cause-present trials). Moreover, we also presented this information summarised with an almost empty bar right before participants had to judge the efficacy of the drug, which would reinforce that overestimation in the number of doses. This is a limitation of our study, and future research should determine whether this distortion in the doses recall is related to the effect of the gross price of each dose, or to the impact of the price relative to a given budget (in our experiment, represented visually by using a budget bar). What seems to be critical, according to the evidence in the causality learning field, is that the distorting memory of having seen more trials in which the drug was administered could establish a critical difference in terms of contingency learning between the expensive and the inexpensive group. People tend to infer causality from coincidences, giving special weight to the probability with which the effect occurs in the presence of the cause (Kao & Wasserman, 1993). Therefore, an illusory overestimation of the probability of the cause, like the one in the expensive drug group, would inflate the perceived contingency, inducing a larger illusion of causality as supported by experimental evidence (Blanco et al., 2014) and simulations of the Rescorla–Wagner model (Vadillo et al., 2016).

Taken together, our results showed that the price of a non-effective drug modulates its perceived efficacy in an experimentally controlled scenario (with the probabilities of the cause and the effect controlled). This suggests that the larger illusion of causality produced by the more expensive treatments might be on the basis of the greater effectiveness of the more expensive placebos. Moreover, this effect of the price in the drug’s effectiveness seems to be mediated by an overestimation of cause-present trials; therefore, the price–quality heuristic seems to influence the decision-making process through a retrospective estimation of the probability of the cause.

Although our results were obtained in a sample of general population rather than from doctors or medical experts, the literature on cognitive biases suggests that there is no reason to think that experts would behave differently (Neal et al., 2022) even in real situations (Frotvedt et al., 2020), particularly in certain conditions such as time pressure (Kelemen et al., 2013), which is a common situation for real-life medical decisions. Thus, similar cognitive factors influence decisions by novices and experts in the health area (Kim & Ahn, 2002; Marsh et al., 2016, 2020), and sometimes experts can even show an exacerbated bias the longer they have been in the profession (Mamede et al., 2010). Moreover, this effect of previous knowledge has been also reported in the field of causal learning, actually inflating the very same type of illusion that we have reported in this paper (Yarritu & Matute, 2015). Therefore, although future research would help to shed light on this issue, the evidence so far suggests that experts may experience the same influence of a drug's price. Understanding the heuristics that people use in medical decision-making could help us to develop strategies to improve such decisions not only for the general population, but also for medical staff who usually has to make tough decisions that have a critical impact on other people’s lives. If, as our results suggest, people use price as a shortcut to infer a drug's efficacy, then successful public health strategies should probably consider educating the public on cognitive biases that affect consumer choices, including this price–quality heuristic in the medical decision-making domain. Also, adopting preventive policies for health professionals in particular, for instance, blinding them to the costs of medication when they have to assess its efficacy, should also reduce the illusion of causality that our results suggest is enhanced by the economic price of medication.

## Data Availability

The materials and data sets generated during and/or analysed during the current study are available in the Open Science Framework repository, https://osf.io/xh8f9/.
